# Linking biosurface reactivity and photosynthesis to investigate *Sphagnum* mosses as ecosystem engineers

**DOI:** 10.1111/nph.71447

**Published:** 2026-08-07

**Authors:** Anna Di Palma, Emanuele Pallozzi, Aridane G. González, Oleg S. Pokrovsky, Ralf Reski, Janice M. Glime, Fiore Capozzi, Norbert Kavasi, Carlo Calfapietra

**Affiliations:** ^1^ Research Institute on Terrestrial Ecosystems (IRET) National Research Council (CNR) Strada Provinciale 35d, n. 9 00010 Montelibretti, (RM) Italy; ^2^ National Biodiversity Future Center (NBFC) Palermo Italy; ^3^ Instituto de Oceanografía y Cambio Global (IOCAG) Universidad de Las Palmas de Gran Canaria (ULPGC) Parque Científico Tecnológico Marino de Taliarte, s/n 35214 Telde Las Palmas Spain; ^4^ GET UMR 5563 CNRS University of Toulouse 14 Avenue Edouard Belin 31400 Toulouse France; ^5^ BIO‐GEO‐CLIM Laboratory Tomsk State University 36 Lenina Ave Tomsk 634004 Russia; ^6^ Plant Biotechnology, Faculty of Biology University of Freiburg Schaenzlestr. 1 79104 Freiburg Germany; ^7^ CIBSS – Centre for Integrative Biological Signalling Studies University of Freiburg Schaenzlestr. 18 79104 Freiburg Germany; ^8^ Department of Biological Sciences Michigan Technological University 219 Hubbell St. Houghton MI 49931 USA; ^9^ Department of Biology University of Napoli Federico II Campus Monte S. Angelo, Via Cinthia 4 80126 Napoli Italy; ^10^ Regional Environmental Co‐Creation Unit Fukushima Institute for Research, Education and Innovation 960‐1295 Fukushima Japan; ^11^ Research Institute on Terrestrial Ecosystems (IRET), National Research Council (CNR) c/o Villa Paolina Via Guglielmo Marconi, 2 05010 Porano (TR) Italy

**Keywords:** abiotic stress, adsorption, climate change, gas exchanges, nature‐based solutions, peat moss, surface functional groups

## Abstract

- *Sphagnum* mosses regulate peatland carbon storage, hydrology, nutrient cycling, and ecosystem functioning. However, the links among their surface chemistry, photosynthesis, responses to submersion and pH remain poorly understood.
- We quantified surface‐chemical properties and biosorption potential in 20 field‐collected species and four axenically cultivated conspecific clones. We measured gas exchange and Chl fluorescence in five representative species under submerged conditions across pH gradients.
- All *Sphagnum* species shared basic surface‐chemical characteristics but differed in surface charge and abundance of reactive sites involved in proton and gas exchange, with generally higher biosorption potential in the *Acutifolia* and *Sphagnum* subgenera. Photosynthesis was maintained under waterlogging, although it varied among species and pH conditions. Reactive surface groups enhanced CO_2_ assimilation. *Sphagnum palustre* showed the broadest physiological tolerance, maintaining stable photosynthesis and photoprotection across all pH levels. *In vitro* cultivation reduced chemical variability among species but preserved the main surface‐chemical properties.
- These results demonstrate that surface chemistry contributes to species‐specific photosynthetic responses and ecological strategies under variable environmental conditions, while supporting the use of *Sphagnum* clones as standardized models for physiological research and environmental applications.

## Graphical abstract

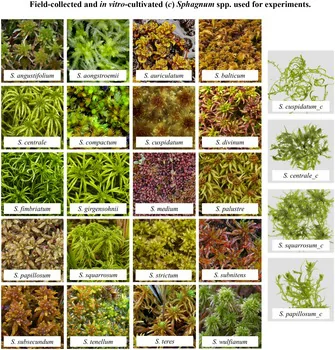

## Resumen

- Los musgos del género *Sphagnum* regulan el almacenamiento de carbono en las turberas, la hidrología, el ciclo de nutrientes y el funcionamiento de estos ecosistemas. Sin embargo, las relaciones entre la química de su superficie, la fotosíntesis y sus respuestas a la inmersión completa en agua y al pH siguen siendo poco conocidas.
- Cuantificamos las propiedades químicas superficiales y el potencial de bioadsorción en 20 especies recolectadas en campo y en cuatro clones de la misma especie cultivados axénicamente. Además, medimos el intercambio gaseoso y la fluorescencia de la clorofila en cinco especies representativas en condiciones de inmersión completa en aguaa lo largo de un gradiente de pH.
- Todas las especies de *Sphagnum* compartieron características químicas superficiales básicas, aunque difirieron en la carga superficial y en la abundancia de sitios reactivos implicados en el intercambio de protones y gases, observándose, en general, un mayor potencial de bioadsorción en los subgéneros *Acutifolia* y *Sphagnum*. La fotosíntesis se mantuvo bajo condiciones de inmersión en agua, aunque varió entre especies y según el pH. Los grupos funcionales reactivos de la superficie favorecieron la asimilación de CO_2_. *Sphagnum* palustre mostró la mayor tolerancia fisiológica, manteniendo una fotosíntesis estable y una fotoprotección eficaz en todo el rango de pH evaluado. El cultivo *in vitro* redujo la variabilidad química entre especies, pero conservó las principales propiedades químicas de la superficie.
- Estos resultados demuestran que la química superficial contribuye a las respuestas fotosintéticas específicas de cada especie y a sus estrategias ecológicas frente a condiciones ambientales variables, al tiempo que respaldan el uso de clones de *Sphagnum* como modelos estandarizados para la investigación fisiológica y las aplicaciones ambientales.

## Introduction

*Sphagnum* mosses have experienced 350 million years of evolution separate from all other mosses (Lueth & Reski, 2023) and play key roles in carbon cycling. They dominate peatlands, which store *c*. 30% of global soil carbon as peat formed via CO_2_ photosynthetic fixation (Serk *et al*., 2021; Liu *et al*., 2024; Minasny *et al*., 2024). High cation‐exchange capacity allows *Sphagnum* to create acidic and nutrient‐poor environments, fostering waterlogged, anoxic conditions that promote peat accumulation (Clymo & Hayward, 1982; Turetsky *et al*., 2012; Rydin & Jeglum, 2013), making peatlands long‐term carbon sinks (Limpens *et al*., 2008; Page *et al*., 2011; Yu *et al*., 2021).

Climate changes and anthropogenic disturbances may accelerate peat decomposition, releasing CO_2_ and CH_4_ and shifting these ecosystems from carbon sink to carbon source (Dise, 2009; Bridgham *et al*., 2013; Charman *et al*., 2013; Joosten, 2015; Bragazza *et al*., 2016; Hopple *et al*., 2020; Loisel *et al*., 2021; UNEP, 2022; Ofiti *et al*., 2023). This amplifies climate warming (Rafat *et al*., 2021), underscoring the urgency of peatland conservation and restoration (Andersen *et al*., 2017; Humpenöder *et al*., 2020; Doelman *et al*., 2023). *Sphagnum*‐based approaches, including *Sphagnum* farming and axenic cultivation (Gaudig *et al*., 2014; Parsons *et al*., 2025), are promising nature‐based solutions, offering an alternative to conventional peat extraction while maintaining ecosystem functions (Temmink *et al*., 2024).

Through secondary metabolite production, nutrient filtering and exceptional water retention capability, *Sphagnum* mosses are ‘ecosystem engineers’ that shape plant communities and hydrological functioning of peatlands (Clymo & Hayward, 1982; Rydin *et al*., 2006; Chiapusio *et al*., 2013; Hamard *et al*., 2019; Liu *et al*., 2020; Gauthier *et al*., 2022; Sytiuk *et al*., 2023; van de Koot *et al*., 2024). Moreover, *Sphagnum* cell surfaces possess chemical reactive sites, such as carboxyl, phosphodiester, phosphoryl, and amino functional groups, for ion binding, proton exchange, and dissolved inorganic carbon speciation at the moss–water interface (González & Pokrovsky, 2014; González *et al*., 2017; Pokrovsky *et al*., 2018). This surface chemistry influences buffering capacity, nutrient cycling (Clymo, 1964) and potentially CO_2_ availability for photosynthesis, and underlies the use of *Sphagnum* moss in environmental monitoring, pollutant biosorption, and reconstructions of atmospheric depositions and climate changes (Shotyk *et al*., 2015; Davies *et al*., 2018; Di Palma *et al*., 2019, 2022).

Because the abundance and composition of surface functional groups vary among mosses (González *et al*., 2017), physicochemical differences between *Sphagnum* species are expected to underpin species‐specific microenvironmental chemistry, with consequences for carbon acquisition, pollutant binding, and ecological role (Clymo, 1973; Rydin *et al*., 2006; Rydin & Jeglum, 2013).

Nevertheless, *Sphagnum* cell surface chemistry and its link to photosynthesis remain underexplored. While research has focused on peatland hydrology and carbon fluxes, proton‐exchange capacity, pH regulation, and photosynthetic efficiency, processes crucial for carbon fixation and pollutant uptake, have not been systematically addressed. This gap is particularly relevant given the lack of stomata in *Sphagnum* photosynthetic tissues (McAdam *et al*., 2021) so that CO_2_ uptake relies on passive diffusion across hydrated surfaces, analogous to mesophyll conductance in vascular plants (Shi *et al*., 2021). Consequently, carbon assimilation in *Sphagnum* is diffusion‐limited, making photosynthetic uptake strongly dependent on water saturation and surface‐mediated chemical processes that regulate pH and CO_2_ speciation in the microenvironment.

Although the effects of tissue water content and drought have been studied (Schipperges & Rydin, 1998; Harris, 2008; Bengtsson *et al*., 2016; Jassey & Signarbieux, 2019; Keane *et al*., 2025), the combined influence of pH and water submersion on *Sphagnum* photosynthesis remains underexplored. Trait‐based and large‐scale ecological studies indicate that water table depth and pH influence species distributions and growth patterns (Bengtsson *et al*., 2016, 2020). However, these studies do not resolve the mechanistic role of surface chemistry in regulating CO_2_ uptake under water‐saturated conditions.

Here, we combine acid–base titration with photosynthetic measurements to elucidate surface reactivity of *Sphagnum* species, and their physiological responses under simulated water saturation. This allows testing whether surface chemistry regulates carbon uptake and biosorption capacity. We quantified biosorption potential, buffering capacity, and functional‐group composition in 20 species collected from nature grown under controlled conditions and in four conspecific species, axenically cloned in bioreactors (Beike *et al*., 2015; Heck *et al*., 2021). Because axenic cultivation is increasingly used in experimental research, biomass production, and peatland restoration (Parsons *et al*., 2025), comparisons with clones will test whether axenic cultivation preserves surface‐chemical traits and whether clones reproduce the physiological and surface‐chemical traits of field‐collected species, informing the validity of laboratory cultivated mosses as models for experimental and applied uses.

Acid–base titration provides an integrative measure of moss surface reactivity by quantifying density and protonation of cell‐wall functional groups across environmentally relevant pH levels. Parameters derived from titration curves, including surface charge excess, site density, and point of zero charge (pH_pzc_; values of pH corresponding to zero net proton adsorption), describe the capacity of *Sphagnum* tissues to exchange cations and buffer pH in the microenvironment, supporting key ecological functions, such as acidification, nutrient retention, inhibition of microbial‐driven decomposition, and carbon cycling.

We assessed gas exchange and Chl fluorescence in five *Sphagnum* species, spanning the major taxonomic sections, using submerged conditions across a pH gradient and an innovative aquatic chamber that integrates accurate gas‐exchange measurements with Chl fluorescence within a highly controlled environment (Hupp *et al*., 2021). This system overcomes the limitations of proxy‐based methods (e.g. O_2_ evolution, fluorescence, and ^14^C incorporation; Pedersen *et al*., 2013). Such equipment has been used for algae (Hupp *et al*., 2021; Steensma *et al*., 2025) and lichens (Meyer *et al*., 2024), and only once for mosses (Norby *et al*., 2023), but analysing samples immediately after field collection and without examining pH effects on photosynthesis or standardized protocols.

We hypothesized that interspecific differences in cell surface chemistry underlie species‐specific variation in microenvironment acidification, biosorption capacity, and CO_2_ acquisition via proton exchange and boundary‐layer chemistry. We further hypothesized that axenic cultivation preserves core surface‐chemical characteristics of natural populations. Specifically, we address the following questions:

1. Is *Sphagnum* photosynthetically efficient under waterlogging?
2. Do species differ in photosynthetic performance and surface chemistry?
3. Are surface‐chemical properties predictive of photosynthetic capacity?
4. Which species exhibit the highest potential for metal biosorption, and thus the highest suitability for biomonitoring and pollutant‐removal applications?
5. Do field‐collected *Sphagnum* and axenic clones differ in their surface‐chemical properties?

## Materials and Methods

### Moss samples

**Fig. 1:**
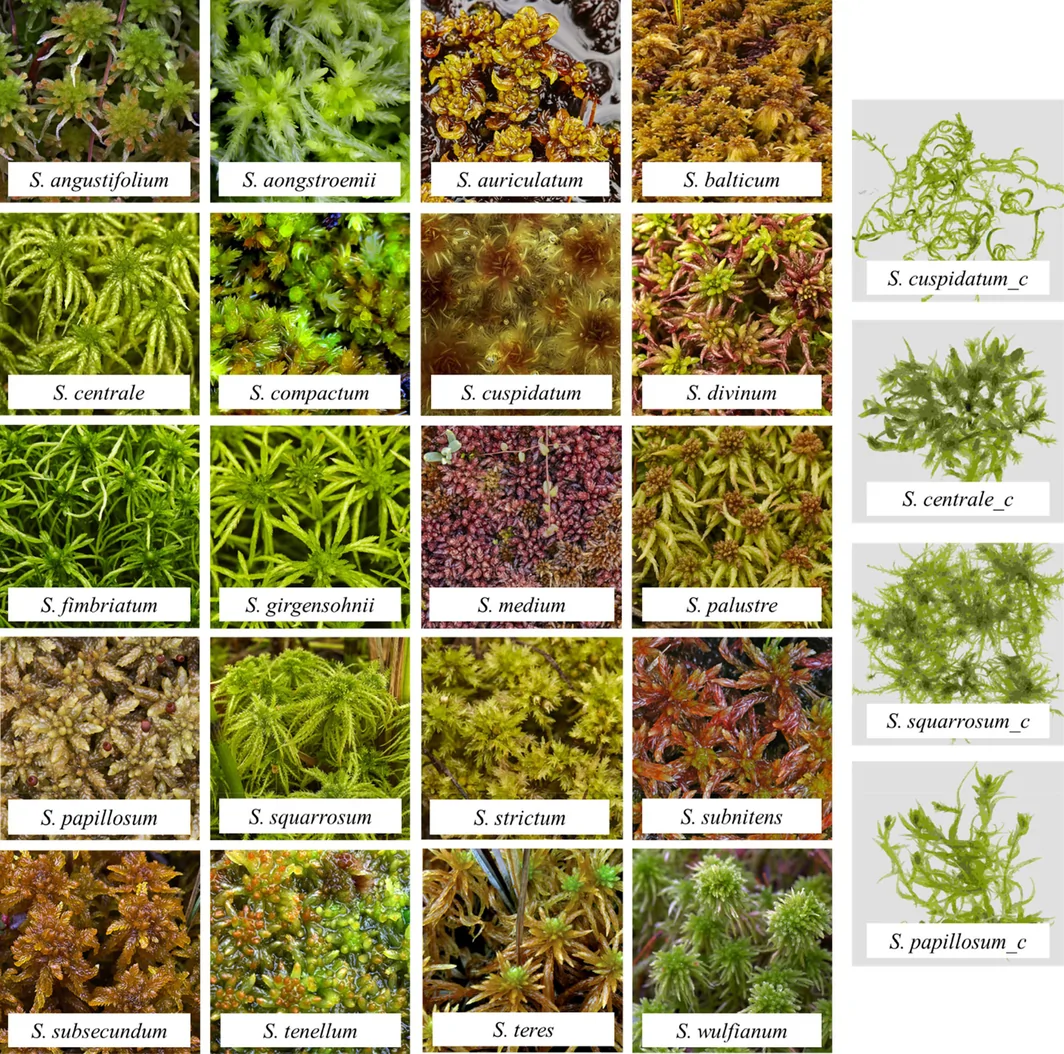
Habit photographs of field‐collected and *in vitro*‐cultivated (*c*) *Sphagnum* spp. used for experiments.

Twenty *Sphagnum* species were investigated (Fig. 1), covering five subgenera. All species were collected in Finland, with the exception of *S. strictum* Sull. (Sweden; Supporting Information Table S1) and identified through dichotomous keys (Laine *et al*., 2018; Michaelis, 2019). Nomenclature follows Shaw *et al*. (2016).

About 10 moss apices (*c*. 2 cm) were cut from shoots and transferred onto a substrate composed of peat, overlaid with commercially available dead *Sphagnum* (Biomeexotic®). Cultures were grown in a climatic chamber (Sanyo‐Gallenkamp SGC970, Loughborough, UK) at 20°C, 16 h : 8 h, light : dark, under nonaturating photosynthetic photon flux density (80 μmol m^−2^ s^−1^), to limit interspecific differences in light acclimation, while avoiding bleaching, etiolation, and photoinhibition. This irradiance represents a compromise based on typical experimental levels (Beike *et al*., 2015; Hao & Chu, 2021; Veeger *et al*., 2025) and field ranges for bryophytes (50–150 μmol m^−2^ s^−1^). Moisture was maintained by covering cultures with transparent lids and regularly misting with deionized water. Additional hydration was ensured by placing perforated polystyrene trays containing the cultures in plastic containers partially filled with deionized water, allowing capillary uptake. All analyses were conducted on newly developed shoots regenerated from the sampled apices after 30–60 d of growth. Additionally, samples of *S. centrale* C.E.O. Jensen, *S. cuspidatum* Ehrh. ex Hoffm., *S. papillosum* Lindb., and *S. squarrosum* Crome cloned in sterile and axenic conditions (Heck *et al*., 2021) were used. These clones were cultivated from spores in photobioreactors in liquid Knop medium (Reski & Abel, 1985) with addition of micronutrients, 2% sucrose and ammonium nitrate (Beike *et al*., 2015). Photoperiod was 16 h : 8 h, with light intensity of 120 μmol m^−2^ s^−1^ (Hohe & Reski, 2005). The pH within bioreactors was adjusted by automatic titration; air was insufflated with 0.3 vvm (Hohe & Reski, 2005).

For each measurement, three to five newly produced, immature shoots were used, each consisting of a shoot apex and a 3‐cm‐long stem. Shoots were selected before the development of characteristic apex morphology and pigmentation to standardize developmental stage and minimize variation in surface‐chemical and physiological traits. Samples exhibiting bleaching or deviating pigmentation were avoided (Maseyk *et al*., 1999).

### Surface characterization

Moss surfaces were characterized in terms of surface charge excess and binding sites by acid–base titration at pH 3–10 (González & Pokrovsky, 2014). Experiments were carried out in 0.01 M NaNO_3_; moss biomass was 1 g_dw_ l^−1^, at RT (20 ± 1°C), under N_2_ and continuous stirring. The pH was measured by a pH/mV meter ion analyser (Orion Lab Star PH111; Thermo Fisher Scientific, Waltham, MA, USA) with an accuracy of ±0.005. Acid and basic titrations were conducted in triplicate for each moss species, via separately adding aliquots of HCl and NaOH titrant solutions (0.01 to 0.1 N). Reference solutions (biomass‐free supernatant solution obtained after 1 h stirring under N_2_ bubbling) were titrated in parallel and used for calculation of charge excess values (González & Pokrovsky, 2014; González *et al*., 2016). The four moss species cultivated in bioreactors were treated in the same way as the conspecific mosses grown in the climatic chamber. Before titration, samples were washed 3 times (10 min each) with filter‐sterilized water (18 MW; 1 g moss/1 l water) to remove excess of dissolved organic carbon (DOC) that could interfere with proton and hydroxyl consumption (Di Palma *et al*., 2019).

### CO_2_ exchange and Chl fluorescence

Gas‐exchange measurements were carried out using a LI‐6800 Portable Photosynthesis Systems (LICOR Biosciences, Lincoln, USA) equipped with a 6800‐18 Aquatic Chamber, a novel setup which enables simultaneous measurement of Chl fluorescence and CO_2_ exchange in wet samples. Before experiments, moss samples were tested at variable degrees of moisture to check performances of the aquatic chamber, particularly its humidity and temperature regulation. Preliminary light‐response curves were performed to define optimal measurement conditions.

One species was randomly selected from each of the five subgenera, that is, *Sphagnum auriculatum* Schimp. (*Subsecunda*), *S*. *balticum* (Russow) C.E.O. Jensen (*Cuspidata*), *S. girgensohnii* Russow. (*Acutifolia*), *S. palustre* L. (*Sphagnum*), and *S. strictum* (*Rigida*). *Sphagnum* samples were analysed while suspended in 15 ml of filter‐sterilized water (18 MW) to assess photosynthetic capacity under submersion. Measurements were conducted as a function of light intensity and pH, with five replicates per species. Eleven light intensities (photosynthetically active radiation (PAR) – ranging from 0 to 1000 μmol m^−2^ s^−1^) and four pH values (3, 5, 7, and 9) were tested. Air temperature was set to 20°C, air flow at 200 μmol s^−1^, and CO_2_ concentration maintained at 430 ppm. Light intensities for generating A/Q response curves were 20, 40, 60, 80, 100, 200, 300, 400, 600, 800, and 1000 μmol m^−2^ s^−1^. Chl fluorescence was assessed simultaneously with gas‐exchange measurements to obtain maximum efficiency of Photosystem II (PSII) photochemistry in light‐adapted state (*F*_v_′/*F*_m_′) at each light level. By contrast, *F*_v_/*F*_m_ (maximum efficiency of PSII photochemistry in dark‐adapted state) was obtained at the beginning of each experiment following 30 min of dark acclimation (Jassey & Signarbieux, 2019). Nonphotochemical quenching (NPQ), the photoprotective mechanism dissipating light energy as heat (Van Gaalen *et al*., 2007; Lu *et al*., 2022), was calculated by the software embedded in the LI‐6800 system. Maximum photosynthetic rate (*A*_max_), quantum yield of CO_2_ assimilation (*Φ*), dark respiration (*R*_d_), and light compensation point (LCP) were estimated from individual A/PPFD (Chang *et al*., 2017).

Solution pH was adjusted by adding aliquots of HCl or NaOH 0.1–0.01 M, and monitored during the experiment. pH oscillations during measurement time of 40 min were minimal, with maximum variation of ±0.4 found only in one replicate.

### Data treatment

Data from titration analyses and gas‐exchange measurements were reported on a dry weight basis. Data analysis and statistical analysis were performed by using Microsoft Excel‐XLSTAT, Synergy Kaleidagraph v.4.0, R statistical environment (v. 4.6, R Core Team), and MATLAB R2024b Update 6.

Normality and homogeneity of variance were assessed using Shapiro–Wilk and Levene tests, respectively. When deviations from normality were detected, data were log‐transformed, appropriate for right‐skewed, strictly positive ecological variables, to stabilize variance while preserving relative differences among observations. Normality was re‐evaluated after transformation using Shapiro–Wilk tests and inspection of *Q*–*Q* plots, and transformed data were used when assumptions were met. ANCOVA was applied for titration and fluorescence measurements, with moss species/section as a fixed factor, and pH (for titration) or light (for fluorescence) as continuous covariate. *Post hoc* comparisons among species/sections within each subgenus were performed using Tukey's test. The Mann–Whitney *U*‐test was used to compare mosses cultivated on peat soil and clones, while the Kruskal–Wallis with Dunn multiple comparison test and Bonferroni correction assessed the significance of light parameter differences as a function of pH and moss species.

Titration curve data were analysed using hierarchical cluster analysis (HCA) to assess similarities among *Sphagnum* species based on surface chemistry. Before clustering, all variables were standardized using *Z*‐score normalization to remove scale effects. Pairwise dissimilarities were calculated using Euclidean distance, and clustering was performed using Ward's minimum variance method (Ward linkage). The optimal number of clusters was explored using the Elbow Method (Within‐cluster Sum of Squares, WSS).

To test the predictive role of chemical reactivity on *Sphagnum* photosynthetic capacity, Pearson correlation analysis was used as an exploratory approach, followed by a GLM. When significant effects were detected, pairwise comparisons among treatment combinations were performed using Tukey's Honestly Significant Difference (HSD) *post hoc* test (*P* < 0.05). *A*_max_, *R*_d_, LCP, and *Φ* were used as dependent variables, while binding sites, pH, and species × pH interaction term were used as predictors. Model explanatory power was assessed using *R* ^2^ values, representing the proportion of variance explained by the fixed predictors.

Surface binding site concentrations and apparent equilibrium constants were estimated using the linear programming model (LPM; Methods S1; Brassard *et al*., 1990; Martinez & Ferris, 2001; Smith & Ferris, 2001), following established protocols (González *et al*., 2016). LPM is based on protonation–deprotonation reactions of surface functional groups, where changes in solution pH are used to calculate net surface charge. By fitting measured and calculated surface charge values, LPM estimates the density of binding sites and their apparent equilibrium constants describing buffering capacity, adsorption potential, and ion‐binding selectivity.

## Results

### Moss surface characterization

**Fig. 2:**
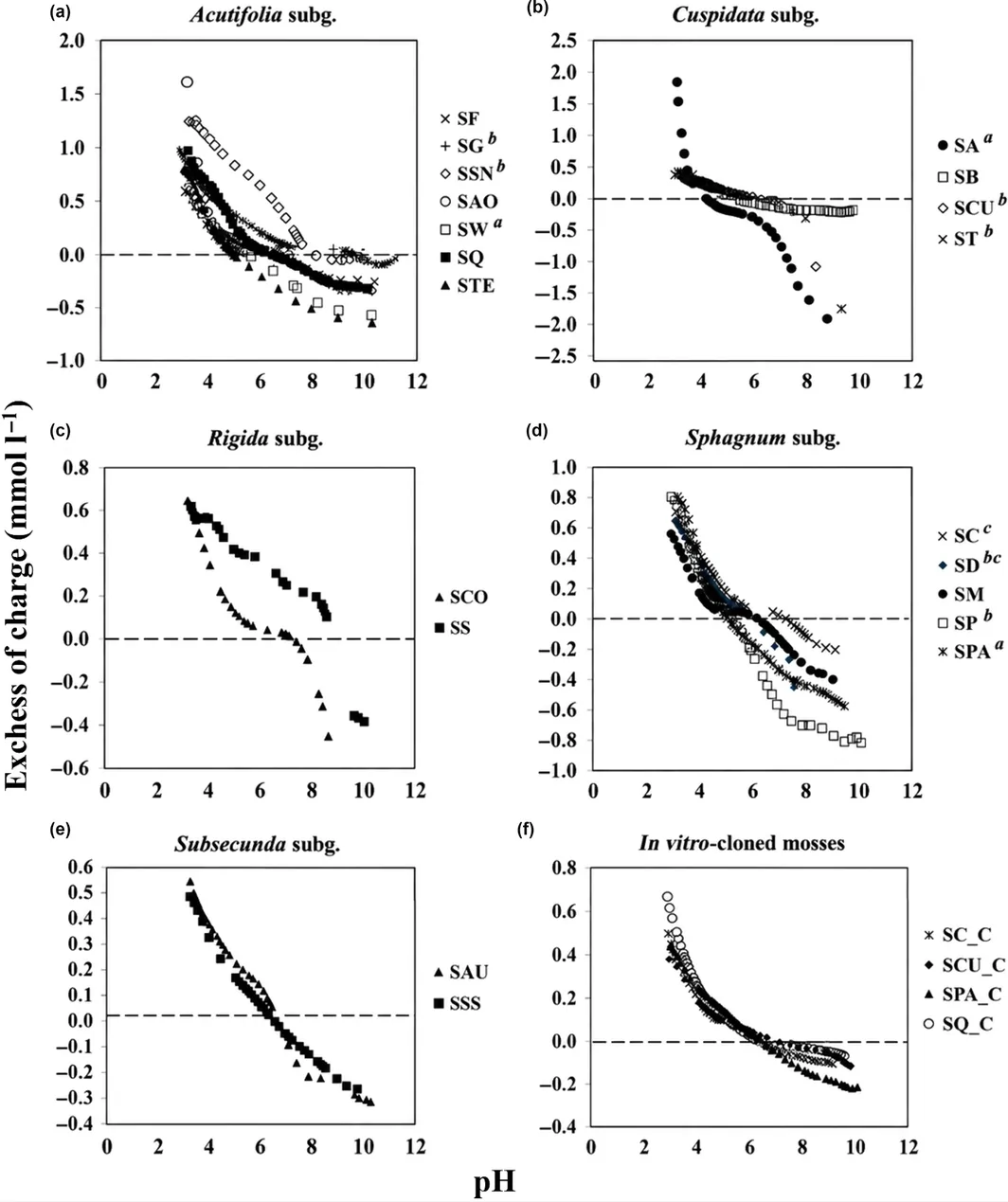
Acid–base titration curves (*n* = 3) revealed interspecific differences in the biosurface chemical properties of field‐collected and *in vitro*‐cultivated *Sphagnum* mosses. Mean titration curves (*n* = 3) are shown for 20 *Sphagnum* species collected from nature (a–e) and 4 *in vitro*‐cloned mosses (f). SA, *S. angustifolium*; SAO, *S*. *aongstroemii*; SAU, *S. auriculatum*; SB, *S. balticum*; SC, *S. centrale*; SCO, *S. compactum*; SCU, *S*. *cuspidatum*; SD, *S. divinum*; SF, *S. fimbriatum*; SG, *S*. *girgensohnii*; SM, *S. medium*; SP, *S. palustre*; SPA, *S*. *papillosum*; SQ, *S*. *squarrosum*; SS, *S*. *strictum*; SSN, *S. subnitens*; SSS, *S. subsecundum*; ST, *S. tenellum*; STE, *S. teres*; SW, *S. wulfianum*; ‘C’, clone. Different letters indicate significant differences among species within each subgenus according to ANCOVA + Tukey's *post hoc* test (*P* < 0.05). Exact *P*‐values: SW vs SG, *P* = 0.0001; SW vs SSN, *P* = 0.0004; SA vs SCU, *P* < 0.0001; SA vs ST, *P* = 0.0002; SPA vs SC, *P* < 0.0001; SPA vs SD, *P* < 0.0001; SPA vs SP, *P* = 0.0002; SP vs SC, *P* = 0.0068.

Surface‐chemical properties of the 20 *Sphagnum* species were described in terms of titration curves (Fig. 2), pH_pzc_, type, and density of surface binding sites (Table 1; Fig. S1).

**Table 1:** Surface acid–base titration and linear programming model (LPM) model results for 20 *Sphagnum* species (*n* = 3).

Subgenus	Section	Species	pH_pzc_	pK_a_	Total BS (mmol g^−1^)	BS (mmol g^−1^)	Functional groups
*Acutifolia*	*Squarrosa*	*S. squarrosum*	5.48	4.45	1.150	0.863	Carboxyl
				7.15		0.287	Phosphoryl
		*S. squarrosum_c*	5.3	3.30	1.001	0.770	Carboxyl/phosphodiester
				5.00		0.099	Carboxyl
				5.85		0.092	Carboxyl
				8.65		0.040	Amine
		*S. teres*	4.61	3.75	1.574	1.079	Carboxyl/phosphodiester
				5.70		0.275	Carboxyl
				6.75		0.220	Phosphoryl
	*Polyclada*	*S. wulfianum*	4.95	3.70	1.267	0.679	Carboxyl/phosphodiester
				5.55		0.239	Carboxyl
				7.00		0.349	Phosphoryl
	*Insulosa*	*S. aongstroemii*	5.63	3.70	1.493	1.413	Carboxyl/phosphodiester
				6.20		0.080	Phosphoryl
	*Acutifolia*	*S. fimbriatum*	5.98	3.75	0.992	0.761	Carboxyl/phosphodiester
				7.15		0.230	Phosphoryl
		*S. girgensohnii*	6.78	3.20	1.539	0.811	Carboxyl/phosphodiester
				4.85		0.195	Carboxyl
				5.85		0.223	Carboxyl
				8.75		0.310	Amine
		*S. subnitens*	6.93	3.90	1.646	0.603	Carboxyl/phosphodiester
				5.55		0.080	Carboxyl
				6.00		0.269	Phosphoryl
				6.95		0.695	Phosphoryl
*Cuspidata*		*S. angustifolium*	4.36	3.60	3.681	2.225	Carboxyl/phosphodiester
				6.30		0.010	Phosphoryl
				7.30		1.446	Phosphoryl
		*S. balticum*	5.31	3.80	0.578	0.147	Carboxyl/phosphodiester
				4.95		0.317	Carboxyl
				6.85		0.095	Phosphoryl
				8.80		0.019	Amine
		*S. cuspidatum*	6.08	3.85	0.502	0.244	Carboxyl/phosphodiester
				4.85		0.140	Carboxyl
				6.25		0.118	Phosphoryl
		*S. cuspidatum_c*	5.22	3.75	0.569	0.371	Carboxyl/phosphodiester
				5.75		0.069	Carboxyl
				6.90		0.062	Phosphoryl
				9.00		0.068	Amine
		*S. tenellum*	5.93	4.15	0.660	0.426	Carboxyl
				6.55		0.234	Phosphoryl
*Rigida*		*S. compactum*	6.19	3.95	1.019	0.734	Carboxyl/phosphodiester
				7.60		0.285	Phosphoryl
		*S. strictum*	8.23	4.45	0.963	0.269	Carboxyl
				6.00		0.090	Phosphoryl
				8.60		0.604	Amine
*Sphagnum*		*S. centrale*	5.74	3.70	1.049	0.488	Carboxyl/phosphodiester
				4.65		0.273	Carboxyl
				7.55		0.288	Phosphoryl
		*S. centrale_c*	5.18	3.60	0.695	0.503	Carboxyl/phosphodiester
				6.25		0.158	Phosphoryl
				8.10		0.035	Amine
		*S. divinum*	5.37	3.95	1.057	0.629	Carboxyl/phosphodiester
				6.45		0.428	Phosphoryl
		*S. medium*	4.63	3.60	1.269	0.799	Carboxyl/phosphodiester
				6.30		0.174	Phosphoryl
				7.25		0.297	Phosphoryl
		*S. palustre*	4.94	3.45	1.952	1.059	Carboxyl/phosphodiester
				5.65		0.248	Carboxyl
				6.25		0.554	Phosphoryl
				8.80		0.092	Amine
		*S. papillosum*	4.79	4.05	1.552	1.038	Carboxyl
				5.30		0.055	Carboxyl
				5.80		0.016	Carboxyl
				6.45		0.292	Phosphoryl
				8.60		0.152	Amine
		*S. papillosum_c*	5.49	3.60	0.606	0.361	Carboxyl/phosphodiester
				5.35		0.073	Carboxyl
				6.35		0.041	Phosphoryl
				7.45		0.076	Phosphoryl
				9.10		0.055	Amine
*Subsecunda*		*S. auriculatum*	6.33	4.00	0.860	0.372	Carboxyl/phosphodiester
				4.75		0.025	Carboxyl
				6.55		0.411	Phosphoryl
				8.90		0.052	Amine
		*S. subsecundum*	6.07	4.00	0.794	0.410	Carboxyl/phosphodiester
				5.80		0.141	Carboxyl
				6.65		0.108	Phosphoryl
				8.00		0.135	Amine

pH_pzc_, pH at point of zero charge; *K*_a_, proton dissociation constant; *c*, moss cloned in photobioreactor; BS, binding sites.

Acid–base properties differed significantly among species within *Acutifolia*, *Cuspidata*, and *Sphagnum* subgenera, whereas no significant differences were detected within *Rigida* or *Subsecunda* (Fig. 2). In *Acutifolia* (Fig. 2a), *S. subnitens* Russow & Warnst. exhibited the highest proton adsorption (1.13 mmol l^−1^), while *S. wulfianum* Girg. showed the most negative surface charge (−0.603 mmol l^−1^). In *Cuspidata* (Fig. 2b), *S. tenellum* (Brid.) Pers. Ex Brid (0.399 mmol l^−1^) had the highest excess of adsorbed protons, while *S. angustifolium* (Russow) C.E.O. Jensen displayed the most negative surface charge (−1.57 mmol l^−1^). Within *Sphagnum* (Fig. 2d), *S*. *papillosum* showed the highest proton adsorption (0.725 mmol l^−1^), whereas *S. palustre* exhibited the most negative surface charge (−0.848 mmol l^−1^) despite similar pH_pzc_ values (Table 1). Overall, interspecific variation in surface charge was generally consistent with differences in pH_pzc_ values (Table 1).

LPM revealed that all *Sphagnum* had surface carboxyl/phosphodiester, carboxyl, phosphoryl, and amino groups, with carboxyl/phosphodiesters dominant and amines generally less abundant (Table 1; Fig. S1). Total binding site density was highest in *Acutifolia* and *Sphagnum* subg. (on average 1.38 mmol g^−1^), intermediate in *Rigida* and *Subsecunda* (0.991 and 0.827 mmol g^−1^, respectively), and lowest in *Cuspidata* (0.435 mmol g^−1^). Carboxyl/phosphodiesters peaked in *Acutifolia* and *Cuspidata* (*c*. 0.88 mmol g^−1^), and were lowest in *Subsecunda* (*c*. 0.39 mmol g^−1^). Carboxyl group abundance was similar across most subgenera (*c*. 0.35 mmol g^−1^) but reduced in *Subsecunda* (0.083 mmol g^−1^), whereas phosphoryls were comparable in *Cuspidata*, *Acutifolia*, and *Sphagnum* (*c*. 0.36 mmol g^−1^) and lower in *Subsecunda* and *Rigida*. Amines were highest in *Rigida* (0.604 mmol g^−1^), and lowest in *Cuspidata* (0.019 mmol g^−1^).

Within *Acutifolia*, carboxyl/phosphodiesters peaked in *S. aongstroemii* C. Hartm. and *S. teres* Ångstr., but were absent in *S*. *squarrosum*, which showed the highest carboxyl content (Table 1). At section level, total binding site amounts were similar, with the highest carboxyl/phosphodiesters content in *Insulosa*, followed by *Squarrosa*, and amines restricted to *S. girgenshonii*. Within *Cuspidata* (Table 1), carboxyl/phosphodiesters were absent in *S. tenellum*, but highest in *S. angustifolium*, which also showed the highest phosphoryl content; amines occurred only in *S*. *balticum*. In *Rigida*, carboxyl/phosphodiesters occurred only in *S. compactum* DC., while carboxyls and amines occurred only in *S. strictum*. In *Sphagnum* (Table 1), carboxyls/phosphodiesters and phosphoryls dominated, peaking in *S. palustre*. Carboxyls were highest in *S. papillosum* and much lower in *S. centrale* and *S. palustre*, while amines were absent in *Sphagnum* subg. All binding sites were present in *Subsecunda* (Table 1), generally higher in *S. subsecundum* Nees. than in *S. auriculatum*, except phosphoryls, which were approximately fourfold higher in the latter species.

**Fig. 3:**
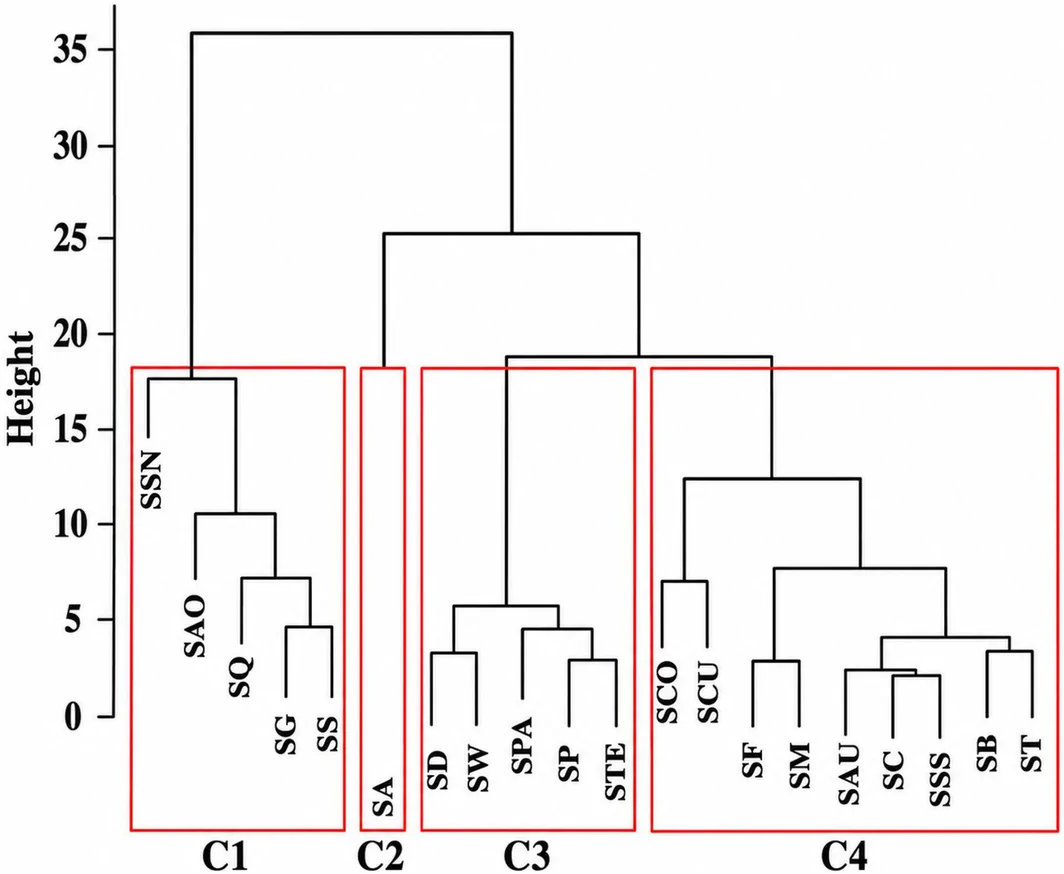
Cluster analysis partially reflected subgeneric and sectional taxonomy. Hierarchical clustering was applied to acid–base titration data (*n* = 3). C1–C4 indicate the four identified clusters. SA, *Sphagnum angustifolium*; SAO, *S*. *aongstroemii*; SAU, *S. auriculatum*; SB, *S. balticum*; SC, *S. centrale*; SCO, *S. compactum*; SCU, *S*. *cuspidatum*; SD, *S. divinum*; SF, *S. fimbriatum*; SG, *S*. *girgensohnii*; SM, *S. medium*; SP, *S. palustre*; SPA, *S*. *papillosum*; SQ, *S*. *squarrosum*; SS, *S*. *strictum*; SSN, *S. subnitens*; SSS, *S. subsecundum*; ST, *S. tenellum*; STE, *S. teres*; SW, *S. wulfianum*.

Cluster analysis showed overall variability in acid–base properties across subgenera, identifying four main clusters (C1–C4; Fig. 3).

Clusters partially reflected subgeneric and sectional taxonomy. Both *Subsecunda* species grouped in C4, while *Sphagnum* species were distributed between C3 and C4. *Acutifolia* displayed partial clustering by section, whereas for *Cuspidata* and *Rigida*, there was significant intra‐group divergence. *Cuspidata* spanned C2 and C4, with *S. angustifolium* forming C2 alone, while *Rigida* was split between C1 (*S. strictum*) and C4 (*S. compactum*).

### Clones vs peat‐grown mosses

Clones grown in photobioreactors displayed a narrow pH_pzc_ range (5.2–5.5; Table 1). Among clones, *S. squarrosum* showed the highest proton adsorption (5.49 mmol l^−1^), and *S. papillosum* the highest negative surface charge (−3.22 mmol l^−1^; Fig. 2f). Compared with clones, peat‐grown *S. centrale*, *S. cuspidatum*, *S. papillosum*, and *S. squarrosum* displayed higher proton adsorption and negative surface charge over comparable pH (often *c*. twofold higher), although pH_pzc_ values were similar for *S. centrale* and *S. squarrosum*, and differed by *c*. 1 pH unit for *S. papillosum* and *S. cuspidatum* (Table 1). Nevertheless, the Mann–Whitney U‐test revealed no significant differences in reactivity among clones, nor between peat‐grown mosses and their conspecific clones.

Total binding site content in clones was highest in *S. squarrosum*, followed by *S. centrale*, *S. papillosum*, and *S. cuspidatum* (Table 1). Peat‐grown mosses generally displayed approximately twofold higher binding site density than their conspecific clones, except *S. cuspidatum*, which had slightly higher values in clones. Among clones, *S. squarrosum* had the highest number of carboxyls/phosphodiesters and carboxyls, while phosphoryls and amines peaked in *S. centrale* and *S. cuspidatum*.

Considering individual binding sites (Table 1), carboxyls/phosphodiesters were slightly higher in *S. centrale* and *S. cuspidatum* clones than in peat‐grown mosses, while carboxyls were absent in cloned *S. centrale* and lower in cloned *S. cuspidatum* compared with peat‐grown mosses, which showed 2‐ to 15‐fold higher amounts in *S. papillosum*, *S. squarrosum*, and *S. cuspidatum*. Phosphoryls, absent in cloned *S. squarrosum*, were about twice as abundant in peat‐cultivated mosses than in clones. Amines were present only in clones, ranging from 0.035 mmol g^−1^ (*S. centrale*) to 0.068 mmol g^−1^ (*S. cuspidatum*).

### Gas exchange as a function of pH

**Fig. 4:**
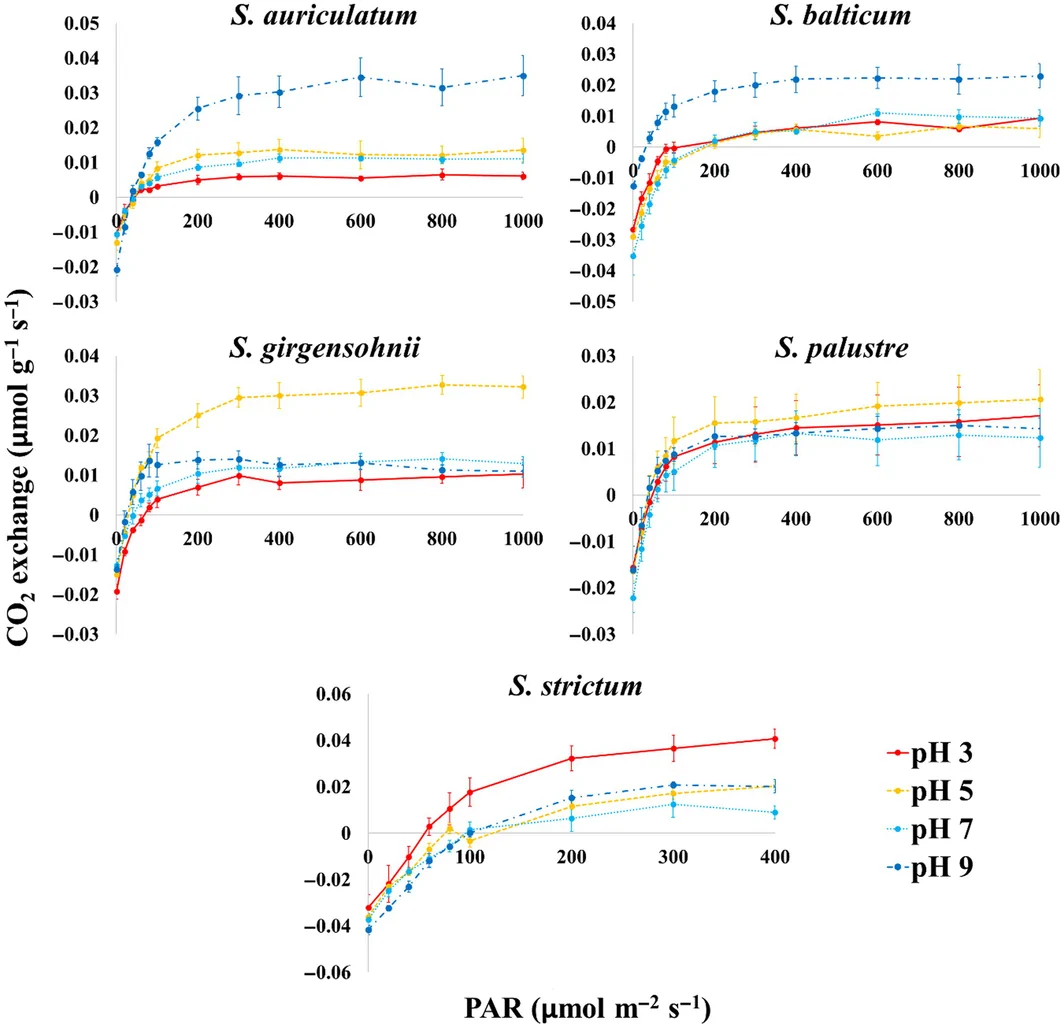
Photosynthetic light‐response curves (*n* = 5) for five field‐collected *Sphagnum* species, shown as CO_2_ exchange rates (nmol g^−1^ s^−1^ ± SE) as a function of photosynthetically active radiation (PAR) (μmol m^−2^ s^−1^), and four pH values.

Gas exchange and Chl fluorescence measurements revealed significant interspecific and pH‐dependent differences in CO_2_ assimilation among the five tested species (*Sphagnum auriculatum*, *S. balticum*, *S. girgensohnii*, *S. palustre*, and *S. strictum*; Fig. 4).

Light saturation occurred at 100–300 μmol m^−2^ s^−1^ PAR in all species. In *S. auriculatum*, CO_2_ assimilation was strongly pH‐dependent, with maximum rates at pH 9 (*c*. 34 nmol g^−1^ s^−1^ at 1000 μmol m^−2^ s^−1^), and substantially lower values at pH 3 and 7 (≤ 14 nmol g^−1^ s^−1^). Similarly, *S*. *balticum* showed maximal assimilation at pH 9 (*c*. 23 nmol g^−1^ s^−1^), and lower rates under acidic‐neutral conditions, with strong negative values at low light. Contrarily, *S. girgensohnii* reached maximum assimilation at pH 5 (*c*. 32 nmol g^−1^ s^−1^ at saturating light), with lower and comparable rates across other pH levels. *Sphagnum palustre* exhibited uniform responses across pH levels, without a clear optimum. In *S. strictum*, measurements were limited to 400 μmol m^−2^ s^−1^, due to reduced vitality at higher irradiance; CO_2_ assimilation peaked at pH 3 (*c*. 43 at 400 μmol m^−2^ s^−1^) and remained markedly lower at pH 7.

Photosynthetic parameters were calculated from light‐response curves, that is, maximum photosynthetic rate (*A*_max_), apparent quantum yield (*Φ*), LCP, and dark respiration rate (*R*_d_; Table 2).

**Table 2:** Photosynthesis parameters calculated from light‐response curves and dark‐adapted maximum quantum yield of PSII (*F*_v_/*F*_m_) for five field‐collected *Sphagnum* species and four pH values.

Species	pH	*A*_max_ nmol g^−1^ s^−1^	*Φ* (×10^−4^) mol mol^−1^	LCP μmol mol^−1^	*R*_d_ μmol g^−1^ s^−1^	*F*_v_/*F*_m_
*S. auriculatum*	3	8.80 ± 2.86^b^	1.41 ± 0.36	47.36 ± 6.89	−6.60 ± 2.05	0.720 ± 0.028
	5	14.10 ± 3.11^ab^	2.60 ± 0.93	48.93 ± 8.03	−11.38 ± 3.74	0.683 ± 0.009
	7	10.91 ± 0.87^ab^	1.17 ± 0.21	57.36 ± 6.87	−6.82 ± 1.58	0.709 ± 0.005
	9	30.42 ± 5.35^a^	2.80 ± 0.37	41.02 ± 3.94	−11.54 ± 2.21	0.694 ± 0.031
*S. balticum*	3	7.56 ± 1.46^ab^	2.40 ± 0.24	85.07 ± 4.78^ab^	−20.40 ± 2.27^ab^	0.643 ± 0.037
	5	5.45 ± 1.59^b^	2.00 ± 0.32	124.77 ± 18.41^b^	−23.06 ± 2.20^ab^	0.587 ± 0.033
	7	6.82 ± 2.31^ab^	2.60 ± 0.68	123.56 ± 20.27^ab^	−28.08 ± 4.47^b^	0.620 ± 0.025
	9	19.62 ± 4.47^a^	2.20 ± 0.37	49.97 ± 15.37^a^	−10.00 ± 3.00^a^	0.699 ± 0.015
*S. girgensohnii*	3	7.83 ± 2.14^b^	1.44 ± 0.34^ab^	111.27 ± 38.63^b^	−11.26 ± 0.88	0.675^b^ ± 0.025
	5	28.50 ± 3.99^a^	2.80 ± 0.37^ab^	34.13 ± 6.03^ab^	−9.40 ± 1.79	0.697^ab^ ± 0.017
	7	8.84 ± 3.63^ab^	1.02 ± 0.43^b^	45.62 ± 9.63^ab^	−4.22 ± 1.66	0.720^ab^ ± 0.008
	9	19.80 ± 5.11^ab^	3.00 ± 0.32^a^	24.6 ± 8.69^a^	−6.92 ± 2.11	0.828^a^ ± 0.055
*S. palustre*	3	23.62 ± 9.80	2.60 ± 0.68	49.94 ± 18.91	−9.52 ± 1.84	0.753 ± 0.009
	5	25.04 ± 8.78	2.60 ± 0.52	46.28 ± 10.54	−10.12 ± 1.37	0.732 ± 0.038
	7	22.76 ± 10.38	2.80 ± 0.66	65.65 ± 19.66	−13.64 ± 2.00	0.768 ± 0.006
	9	18.88 ± 4.55	2.20 ± 0.37	39.70 ± 8.09	−9.20 ± 3.09	0.774 ± 0.005
*S. strictum*	3	40.65 ± 4.15^a^	5.00 ± 0.95	61.33 ± 10.42	−30.94 ± 7.22	0.582^ab^ ± 0.025
	5	20.31 ± 1.76^ab^	4.80 ± 0.37	71.90 ± 3.39	−34.62 ± 3.33	0.502^b^ ± 0.010
	7	12.40 ± 2.84^b^	3.60 ± 0.68	100.08 ± 16.51	−31.66 ± 1.31	0.611^a^ ± 0.016
	9	20.15 ± 2.93^ab^	4.40 ± 0.24	90.46 ± 4.12	−39.50 ± 1.35	0.588^ab^ ± 0.021

Values (*n* = 5) are given with SE. *A*_max_, maximum photosynthetic rate; Φ, apparent quantum yield; LCP, light compensation point; *R*_d_, dark respiration rate. Different letters indicate significant differences among treatments (*P* = 0.0083) according to Kruskal–Wallis test + Dunn multiple comparison and Bonferroni correction.

Maximum net CO_2_ assimilation (*A*_max_) varied markedly among species and pH levels. The highest *A*_max_ occurred in *S. strictum* at pH 3 and in *S. auriculatum* at pH 9, followed by *S. girgensohnii* and *S. palustre* (pH 5), and *S. balticum* (pH 9). In *S. auriculatum*, *A*_max_ increased with pH, whereas *S. balticum* peaked at pH 9. By contrast, *S. girgensohnii* exhibited maximum *A*_max_ at pH 5, while *S. palustre* showed no significant pH‐dependent variation. *Sphagnum strictum* reached maximum *A*_max_ in acidic conditions, with lower values at neutral pH. Apparent quantum yield (*Φ*) followed species‐specific responses to pH. It remained relatively stable in *S. balticum*, *S. palustre*, and *S. strictum*, whereas *S. girgensohnii* showed significantly higher values at pH 9. The LCP varied significantly with pH in *S. balticum* and *S. girgenshonii*, decreasing under alkaline conditions. By contrast, LCP remained constant across pH levels in *S. auriculatum* and *S. palustre*, while *S. strictum* maintained consistently high values. Respiration rates (*R*_d_) differed among species but were generally unaffected by pH, except for *S. balticum*, whose respiration decreased at pH 9. The lowest *R*_d_ occurred in *S*. *auriculatum*, *S. girgenshonii*, and *S*. *palustre*, whereas *S. strictum* consistently showed the highest values. The maximum quantum efficiency of PSII in dark‐adapted samples (*F*_v_/*F*_m_) exceeded 0.70 in all species and generally declined under acidic conditions. This trend was strongest in *S. girgensohnii*, which reached the highest *F*_v_/*F*_m_ values at pH 9, while *S. strictum* displayed the lowest overall values. In *S. auriculatum*, *S. balticum*, and *S. palustre*, *F*_v_/*F*_m_ remained relatively stable across treatments.

Pearson correlations revealed distinct, pH‐dependent relationships between surface binding sites and photosynthesis parameters (Table S2). Total binding capacity showed weak correlations and explained less variance than individual functional groups. *A*_max_ was positively correlated with carboxyl–phosphodiesters and phosphoryls at pH 5–7, whereas amines were positively correlated only at pH 3. Carboxyls showed weak or negative correlations, becoming strongly negatively correlated to *A*_max_ at pH 9. The quantum yield *Φ* correlated strongly with carboxyl–phosphodiesters at pH 5 and with amines across all pH levels. LCPs were negatively correlated with phosphoryls across all pH levels, while positively correlated with carboxyls at pH 3 and with amines only under alkaline conditions. Strong positive correlations existed between R_d_ and phosphoryls at pH 3–7, moderate with carboxyl–phosphodiesters, and negative with amines.

GLM analysis (Table S3) revealed significant variation in the relationships between surface‐chemical reactivity and photosynthetic parameters across pH treatments. *Sphagnum balticum* and *S. girgenshonii* exhibited clear pH‐dependent shifts in the relationships between surface functional groups and photosynthetic traits. In *S. balticum*, the correlation patterns between *A*_max_ and surface chemistry at pH 9 differed significantly from those observed under acidic and neutral conditions. Similar trends were observed for LCP, with significant shifts under alkaline conditions in *S. balticum* and between pH 3 and 9 in *S. girgenshonii*. For *R*_d_, significant differences among pH treatments were detected only in *S. balticum*, whereas for *Φ* only in *S. girgenshonii*, showing heterogeneous responses in the relationships between quantum yield and surface chemistry across pH conditions. Overall, the strongest explanatory power was observed for *R*_d_ (*r* ^2^ = 0.77), followed by *Φ* (*r* ^2^ = 0.52) ~ LCP (*r* ^2^ = 0.50), and *A*_max_ (*r* ^2^ = 0.43).

NPQ (Fig. S2) increased with light intensity in all species and was pH‐dependent, except in *S. palustre*. Significant differences at pH 9 occurred in *S. auriculatum*, *S. balticum*, and *S. girgensohnii*. *Sphagnum auriculatum* showed no clear additional pH‐specific pattern beyond the strong effect of pH 9, while *S. balticum* and *S. girgensohnii* showed strongest NPQ responses at pH 9, particularly above 400 μmol m^−2^ s^−1^, with additional differences between intermediate pH levels. Contrarily, *S. strictum* exhibited much lower NPQ overall, with a heterogeneous response across pH levels and a clear separation of pH 7 from other treatments. In all species and for all pH values, the maximum efficiency of PSII photochemistry in light‐adapted state (*F*_v_′/*F*_m_′) decreased with increasing light, with a steeper decline within the first 200 μmol m^−2^ s^−1^ PAR. The effect of pH on this trend was generally weak or absent, except in *S. auriculatum*, *S. strictum*, and *S. girgensohnii*, where significant species‐specific pH‐dependent modulation of photochemical efficiency was observed. Namely, in *S. auriculatum* the *F*_v_′/*F*_m_′ ratios showed significant differences between pH 9 and all other pH levels, while in *S. girgensohnii*, they were only between pH 3 and 9. Instead, *S. strictum* showed multiple significant differences with pairwise contrasts distributed across the pH gradient.

## Discussion

### Surface chemistry

The 20 studied *Sphagnum* species showed strongly amphoteric surfaces (i.e. positively or negatively charged depending on pH), controlled by dissociation of carboxyl, phosphodiester, phosphoryl, and amino groups derived from cell‐wall organic compounds (Renault *et al*., 2017; Bengtsson *et al*., 2018; Hodgkins *et al*., 2018; Astolfi *et al*., 2024). Carboxyls and phosphodiesters dominate proton and metal binding (Ringqvist *et al*., 2002; Ringqvist & Öborn, 2002; Vijayaraghavan & Yun, 2008; González & Pokrovsky, 2014; Di Palma *et al*., 2019; Pokrovsky *et al*., 2024), while phosphoryls enhance Ca^2+^/Mg^2+^ binding under circum‐neutral pH, supporting ion selectivity in minerotrophic conditions. Amines broaden ion‐exchange, anion binding and ammonium uptake under extreme pH or nutrient‐limited conditions (Mishra *et al*., 2010). This surface chemistry enables active acidification of the microenvironment by *Sphagnum*, and drives metal adsorption and carbon dynamics (Clymo, 1973; Fein *et al*., 2001; Borrok & Fein, 2004; Gélabert *et al*., 2004). Ion exchange likely occurs by rapid adsorption at both external and internal cell surfaces, followed by slower diffusion (Qin *et al*., 2006), consistent with the lack of a vascular system and the high porosity of *Sphagnum* tissues (Brown, 1982; Reski, 1998).

Our results indicate that surface chemistry underlies both the ecological distribution and biosorption capacity of *Sphagnum* species, potentially enabling them to tolerate and occupy a broader range of ionic conditions than those in which they are typically observed (Table S1). For example, *S. wulfianum*, with low pH_pzc_ and highly negative surface charge, exhibits high cation affinity and potential adaptation to nutrient‐poor, ombrotrophic environments, despite its common occurrence in minerotrophic sites. In *S. palustre*, abundant carboxyl/phosphodiester and carboxyls combined with consistently negative surface charge support strong proton adsorption and metal binding, suggesting good performance in ombrotrophic or acidic minerotrophic habitats, despite its classification as mesotrophic. Contrarily, *S. papillosum*, lacking carboxyl/phosphodiesters, exhibited limited binding versatility, making it better suited to minerotrophic sites with moderate pH and ion‐binding demand (Rydin & Jeglum, 2013). Such patterns support evidence that ionic niche preferences in *Sphagnum* are evolutionarily labile (Granath *et al*., 2010; Johnson *et al*., 2015).

Similarities in chemical profile among *Sphagnum* species indicate subgenus‐level chemical traits conserved from early developmental stages and maintained even in artificial cultivation. Conversely, variation in chemical reactivity among closely related taxa (within *Cuspidata* and *Rigida*) likely reflects biochemical rather than anatomical differences (Limpens *et al*., 2017; Bengtsson *et al*., 2018).

Overall, *Acutifolia* and *Sphagnum* subg. exhibited higher biosorption potential than *Cuspidata*, *Rigida*, and *Subsecunda*, with *S. angustifolium*, *S. subnitens*, and *S. palustre* emerging as the most efficient metal adsorbents among the 20 studied species.

Species of *Acutifolia* showed comparatively high surface reactivity and buffering capacity, consistent with their common occurrence in minerotrophic environments. In particular, *S. subnitens* combined high pH_pzc_ and strong proton adsorption capacity, supporting efficient buffering under elevated base cation concentrations. Similarly, *Sphagnum* subg. was characterized by high site densities dominated by carboxyl/phosphodiester and phosphoryl groups, supporting strong proton and metal binding. The absence of amines across the subgenus further suggests that chemical interactions are prevalently driven by cation exchange.

In *Cuspidata*, *S. angustifolium* emerged as the most chemically reactive, combining strong negative surface charge, high carboxyl/phosphodiester and phosphoryl content, and low pH_pzc_, all indicative of efficient cation binding under neutral to slightly acidic conditions. These properties support both its high biosorption potential and competitiveness in ion‐rich or weakly minerotrophic habitats. Contrarily, *S. cuspidatum* and *S. tenellum* had higher pH_pzc_ and lower site densities, consistent with weaker buffering capacity and adaptation to highly acidic, nutrient‐poor hollows. The exceptional performance of *S. angustifolium* further demonstrates that intra‐subgeneric variability may exceed inter‐subgeneric trends, highlighting the importance of species‐level screening in pollutant biosorption studies.

Contrarily to other subgenera, *Rigida* and *Subsecunda* showed uniform chemical reactivity. Within *Rigida*, *S. compactum* and *S. strictum* showed similar overall charges, but differed in functional‐group composition. *Sphagnum compactum* had carboxyl/phosphodiesters, suggesting greater affinity for multivalent cations, whereas *S. strictum* showed higher pH_pzc_, carboxylates, and amines, favouring amphoteric behaviour and broader metal‐binding capacity under variable pH conditions. These traits align with *S. strictum* preference for alkaline, minerotrophic habitats.

*Subsecunda* species exhibited balanced functional‐group compositions, moderate pH_pzc_, and weak proton adsorption, consistent with their occurrence in transitional oligo‐ to mesotrophic habitats. *Sphagnum subsecundum*, having high total site abundance, possesses broader cation‐exchange capacity and may therefore be better suited to slightly nutrient‐poor minerotrophic conditions, whereas *S. auriculatum* may specialize in binding multivalent cations via phosphoryls. Despite these differences, both had similar pH_pzc_ and surface charges, maintaining negatively charged cell walls across typical peatland pH values, enabling stable cation retention and moderate acidification.

### Moss clones

Despite a few exceptions (absence of phosphoryls in cloned *S. squarrosum* and presence of amines only in clones), baseline functional groups were maintained, suggesting that *in vitro* cultivation preserves core chemical traits of *Sphagnum* tissues. Species‐specific differences in cell‐wall reactivity were evident, although chemical differentiation among clones was generally reduced, likely due to the uniform, axenic growth conditions (Beike *et al*., 2015; Di Palma *et al*., 2017).

Peat‐grown mosses had higher functional‐group abundance and proton adsorption, reflecting enhanced ion‐exchange capacity and buffering under natural conditions, likely influenced by origin of tissues, microbial interactions, and substrate chemistry. Peat‐grown shoots develop from existing gametophytic tissue and retain mature morphological and biochemical features, while clones originate from spores, exhibit *de‐novo* tissue formation with less structural complexity and no microbial associations (Kostka *et al*., 2016; Parsons *et al*., 2025). Normalizing site numbers to specific surface area (SSA_BET_), which is roughly twice as high in clones, may account for differences in surface reactivity (González *et al*., 2016).

Overall, homogeneity among clones highlights their potential to reproduce key physicochemical properties of natural populations, providing predictable and reproducible physiological performance. Clones exhibit photochemical efficiency despite the absence of natural environmental variability, likely due to minimized photoinhibition and metabolic disruption by optimal nutrient availability and reduced stress (Schipperges & Rydin, 1998; Keightley *et al*., 2024). Therefore, *Sphagnum* clones are well‐suited for research and applications, including wastewater treatment, biomonitoring, and peatland restoration.

### Photosynthetic capacity

*Sphagnum* mosses maintained photosynthetic activity under waterlogging, but performance varied among species and was influenced by pH. The interspecific and pH‐dependent differences in the five studied species *S. auriculatum*, *S. palustre*, *S. balticum*, *S. girgensohnii*, and *S. strictum* align with their ecological specialization being linked to environmental pH (Harley *et al*., 1989; Haraguchi, 1996; Van Gaalen *et al*., 2007). These differences affect CO_2_ assimilation, light‐use efficiency, photoprotection, and respiration, and presumably species adaptability to variable conditions. For example, *S. strictum* displayed the highest *A*_max_ at pH 3, indicating a physiological adaptation to acidic conditions. Conversely, *S. auriculatum* performed best in alkaline conditions, while *S. palustre* maintained stable *A*_max_ across the entire pH range, indicating broad physiological tolerance. Although *Sphagnum* mosses prefer wet, acidic conditions and are negatively impacted by alkaline waters (Koks *et al*., 2019), *S. auriculatum* is moderately acidophilic (Hugonnot & Chavoutier, 2024), while *S. palustre* occurs in regions with HCO_3_^−^ rich surface water and is cultivated for paludiculture (Koks *et al*., 2025), suggesting ecological advantages as a generalist species. *Sphagnum girgensohnii* exhibited maximum photosynthetic performance at pH 5, contrasting with results of Haraguchi *et al*. (2003), who reported photosynthetic optimum around pH 7.2, although with minimal variation across pH levels. Contrarily, *S. balticum* exhibited low assimilation from acidic to neutral pH values, indicating limited competitive ability and sensitivity to chemical fluctuations.

Photosynthetic saturation in light‐response curves between 100–300 μmol m^−2^ s^−1^ PAR reflected adaptation to shade/low‐light (Harley *et al*., 1989; Davey & Rothery, 1997; Van Gaalen *et al*., 2007). This adaptation varies among species and pH values (Harley *et al*., 1989; Maseyk *et al*., 1999). Here, *S. girgensohnii* showed the highest photosynthesis performance and lowest LCP at pH 5, reflecting efficient carbon assimilation in low light of its natural habitat (Laine *et al*., 2018). Conversely, *S. balticum* exhibited higher LCP values, especially at pH 5 and 7, suggesting stronger light dependence and limited competitiveness under shaded conditions (Van Gaalen *et al*., 2007).

The coupling between carbon assimilation, PSII photochemistry, and photoprotective energy dissipation revealed different strategies of energy regulation among species. *Sphagnum auriculatum* combined high *A*_max_ with elevated NPQ at pH 9, while maintaining relatively stable *F*_v_/*F*_m_ and enhanced *F*_v_′/*F*_m_′ at that pH. This pattern indicates that photoprotection and photochemistry were maintained simultaneously, indicating a highly plastic acclimation strategy under alkaline conditions in which photosynthetic performance is not constrained by energy dissipation. By contrast, *S. girgensohnii* and *S. balticum* showed increased NPQ under pH conditions associated with reduced photosynthetic performance. This pattern, coupled with maintained PSII efficiency, suggests a shift from carbon acquisition towards protection of the photosynthetic apparatus under less favourable chemical conditions. Moderate NPQ combined with high *F*_v_/*F*_m_, such as in *S. girgensohnii* under strongly alkaline conditions, indicates efficient balance between photon use and energy dissipation, allowing continuous carbon assimilation under stress (Korrensalo *et al*., 2017). *Sphagnum strictum*, showed a contrasting pattern. Despite maximum assimilation under acidic conditions, it exhibited the lowest *F*_v_/*F*_m_ values, generally low NPQ, and strong pH‐dependent variation in *F*_v_′/*F*_m_′, indicating reduced photochemical capacity and limited flexibility in photoprotective regulation, with pH affecting PSII photochemistry more strongly than NPQ. Finally, *S. palustre* showed minimal variation in *A*_max_, NPQ, *F*_v_/*F*_m_, and *F*_v_′/*F*_m_′ across the pH gradient, supporting a conservative strategy characterized by stable photosynthetic performance and low sensitivity of both photochemical and photoprotective processes to chemical variation (Keightley *et al*., 2024). Across all species, *F*_v_′/*F*_m_′ declined with increasing PAR, particularly between 0–200 μmol m^−2^ s^−1^, consistent with PSII downregulation in shade‐adapted mosses (Van Gaalen *et al*., 2007; Sepp & Ilomets, 2008). However, significant pH effects were restricted to *S. auriculatum*, *S. girgensohnii*, and *S. strictum*, indicating that pH influences the PSII regulation under light (i.e. when PSII is actively functioning) in a species‐specific manner, whereas PSII structural integrity, reflected by generally high *F*_v_/*F*_m_ values, remained largely preserved.

Quantum efficiency of PSII (*Φ*) was highest in *S. strictum* across all pH levels, despite reduced PSII efficiency, suggesting a trade‐off between net carbon gain and protective energy dissipation. *Sphagnum girgensohnii* showed lower *Φ* coupled with decreased *A*_max_ at pH 3 and 7, while the other mosses showed invariant *Φ* across pH levels, highlighting divergent energy management strategies (Van Gaalen *et al*., 2007). Dark respiration rates (*R*_d_) varied substantially among species, but were largely unaffected by pH, except in *S. balticum*, which showed reduced respiration under alkaline conditions. The elevated respiration rates observed in *S. strictum* and *S. balticum* indicate higher metabolic costs or increased stress‐related carbon losses that limit net assimilation (Robroek *et al*., 2009). In particular, the combination of low *A*_max_ and high *R*_d_ in *S. balticum*, except in alkaline conditions, highlights its limited photosynthetic competitiveness across most pH levels.

Overall, our findings suggest that *S. palustre*, beyond functioning as an efficient ion adsorbent, emerged as the most stress‐tolerant and generalist species, maintaining stable CO_2_ assimilation, PSII function, and photoprotective activity across the entire pH range. Contrarily, the remaining species exhibited more specialized strategies: *S. auriculatum* showed high physiological plasticity and performed best under alkaline conditions, *S. strictum* was adapted to acidic conditions, whereas *S. girgensohnii* and *S. balticum* displayed more dynamic photoprotective responses to changing pH.

Although mosses were grown under nonsaturating irradiance close to the LCP for some, the fact that light saturation was reached well above the growth level, together with steep increases in CO_2_ assimilation at higher light intensities, indicates that growth conditions did not limit photosynthetic capacity or bias the comparative interpretation of photosynthetic performance. Furthermore, all species exhibited light saturation at 100–300 μmol m^−2^ s^−1^, while measurements extended to 1000 μmol m^−2^ s^−1^, allowing full expression of photosynthetic potential. Chl fluorescence measurements confirmed functional and plastic photosystems, with no evidence of chronic or low‐light‐induced impairment, and the achievement of species‐specific *A*_max_ values indicates that comparative patterns reflect intrinsic physiological differences rather than growth‐light acclimation.

Our measurements provided a mechanistic baseline for species‐specific photosynthetic responses, relevant for predicting how *Sphagnum* species may respond to environmental fluctuations, including shifts in water table or pH during flooding events. Although long‐term field responses may differ due to acclimation, biotic interactions, variable nutrient availability, and cumulative stresses, the patterns observed in *A*_max_, *Φ*, LCP, and NPQ reveal intrinsic pH preferences and stress tolerances that are likely to persist in nature. Trends such as the high *A*_max_ of *S. strictum* at low pH, or the broad pH tolerance of *S. palustre*, suggest predictable differences in competitiveness under acidic, neutral, or alkaline conditions in peatlands.

### Chemical reactivity and photosynthetic capacity

#### Surface chemistry influences CO_2_ acquisition beyond pH_pzc_ effects

In water, photosynthesis is limited by slow CO_2_ diffusion (Murray *et al*., 1989; Silvola, 1990; Li *et al*., 1992; Williams & Flanagan, 1996; Van Gaalen *et al*., 2007). Because dissolved organic carbon occurs in a pH‐dependent equilibrium among CO_2_, HCO_3_^−^, and CO_3_^2−^, local pH conditions strongly influence CO_2_ availability (Allen & Spence, 1981; Raven *et al*., 1998). Therefore, CO_2_ uptake depends also on the ability of moss surfaces to modify the moss–water interface.

The point of zero charge (pH_pzc_) determines the protonation state of surface functional groups. At pH values above pH_pzc_, negatively charged carboxyl and phosphoryl groups dominate and can exchange protons with surrounding water, buffering pH within the diffusive boundary layer. This localized buffering, particularly within hyalocysts and around photosynthetically active cells, may shift the CO_2_/HCO_3_^−^ equilibrium towards molecular CO_2_, which diffuses more readily across membranes than bicarbonate (Glime, 2020). Since *Sphagnum* depends largely on passive diffusion for carbon uptake (Waite & Sack, 2011), this mechanism could enhance photosynthetic performance.

Species with low pH_pzc_ (*S*. *angustifolium*, *S*. *medium* Limpr., S. *teres*) are expected to remain negatively charged in a broader pH range, and thus to show greater acidification potential. However, our results indicate that pH_pzc_ alone does not explain photosynthetic responses. For example, *S. auriculatum* and *S. balticum* showed maximum CO_2_ assimilation at pH 9, well above their pH_pzc_ values, whereas *S. strictum* and *S. girgensohnii* performed best at acidic pH values below their pH_pzc_. *Sphagnum palustre* maintained relatively stable assimilation across the pH gradient, indicating broad tolerance rather than a direct pH_pzc_ effect.

#### Surface reactive sites indirectly shape photosynthetic efficiency

Both correlation and GLM analyses revealed strong relationships between surface chemistry and photosynthetic performance, with a significant species‐ and pH‐driven variation particularly for *S. balticum* and *S. girgensohnii*.

Carboxyl–phosphodiester groups appear to play an important role in supporting maximum photosynthetic performance under acidic to circum‐neutral conditions, as indicated by their consistently strong positive associations with *A*_max_ across pH 3–7. Their reduced relationship with *A*_max_ at pH 9 suggests that their contribution becomes less important under strongly alkaline conditions, likely due to shifts in carbonate chemistry. Functionally, phosphodiesters may act synergistically with carboxyl groups by retaining cations and water molecules close to the cell surface, thereby stabilizing hydrated and proton‐enriched microsites created by carboxyls at the moss–water interface (Clymo, 1964; Vijayaraghavan & Yun, 2008).

By contrast, phosphoryl groups showed a different pattern, with weaker or variable correlations with *A*_max_, but strong and consistent positive relationships with dark respiration. This indicates a primary role in regulating respiratory CO_2_ dynamics rather than directly enhancing peak photosynthetic rates. Phosphoryls can bind and release protons (Baker *et al*., 2009), and due to their greater negative charge per unit than carboxyls (Franz, 2001), may stabilize proton‐rich microenvironments generated by respiration. Thus, rather than primarily contributing as CO_2_‐generating sites, phosphoryl‐rich surfaces appear to control the retention of respired CO_2_ within the diffusive boundary layer for its potential reassimilation in moss cells. As the LCP reflects the irradiance at which photosynthesis balances respiration, surfaces promoting local CO_2_ retention are expected to support positive net carbon gain at lower light intensities, resulting in lower LCPs. In this context, the consistently negative relationships between phosphoryls and LCP further indicate an important role in sustaining photosynthetic performance under low irradiance.

Carboxyl groups do not appear to be primary determinants of maximum photosynthetic capacity, as their association with *A*_max_ was weak and inconsistent across pH levels. Instead, their influence is more evident under low irradiance, where positive relationships with LCP at acidic pH suggest a role in modulating carbon balance when light availability is limiting. However, compared with phosphoryls, carboxyl effects on LCP were weaker and mainly restricted to acidic conditions, indicating a more limited contribution to low‐light carbon retention and reassimilation processes.

By contrast, amine‐rich surfaces do not appear to promote maximum carbon assimilation, particularly under neutral to alkaline conditions where their relationships with *A*_max_ remain weak or negative. Near neutral pH, amines occur mainly in their protonated form and may act as basic sites, potentially reducing the pool of CO_2_ by its conversion into bicarbonate and carbonate, particularly at high pH (Jiang *et al*., 2004; Hamdy, 2021). Their positive association with LCP under alkaline conditions further suggests reduced photosynthetic efficiency at low irradiance. At the same time, the consistently negative relationships between amines and dark respiration indicate that amine‐rich surfaces are not closely linked to respiratory carbon recycling processes.

Despite their apparently limited role in CO_2_ retention, amines showed the strongest and most consistent positive relationships to quantum yield (*Φ*) across nearly all pH values. Since *Φ* reflects efficiency of CO_2_ fixation per absorbed photon, this pattern suggests that amines may enhance photon‐use efficiency at low irradiance, likely through mechanisms not directly related to bulk CO_2_ availability (e.g. microenvironmental charge stabilization or indirect support of bicarbonate‐related carbon acquisition).

Taken together, these results demonstrate that surface chemistry influences the photosynthetic responses, primarily through functionally distinct roles of specific groups. Carboxyl–phosphodiester groups are most closely associated with maximum carbon assimilation, phosphoryls with respiratory CO_2_ retention and low‐light carbon balance, and amines with photon‐use efficiency. Overall, these findings support a model in which *Sphagnum* photosynthetic performance is regulated by multiple microscale chemical pathways linked to respiratory CO_2_ retention, low‐light use, and local carbon availability.

#### Surface chemistry underlies ecological strategies and carbon acquisition

At the ecosystem scale, interspecific variation in moss surface chemistry and photosynthetic responses may contribute to species sorting along pH and hydrological gradients, influencing peatland structure and carbon balance. Our results reveal adaptive strategies and trade‐offs among *Sphagnum* species in relation to their natural habitats. Ombrotrophic species are specialized for acidic, nutrient‐poor environments, exhibiting maximal CO_2_ assimilation at low pH, high densities of carboxyls and phosphoryls for microenvironment acidification, and elevated photoprotection under suboptimal conditions. These traits enhance carbon acquisition in acidic waters but limit performance under neutral‐alkaline conditions, reflecting a trade‐off between high specialization and environmental versatility. By contrast, minerotrophic species maintain stable photosynthetic performance across a broader pH range, benefiting from moderate surface chemistry and buffering capacity. These generalist strategies confer greater ecological flexibility, albeit at the cost of peak photosynthetic rates under acidic conditions. From an applied perspective, species with high densities of carboxyls and phosphoryls combine enhanced carbon fixation with high biosorption potential, making them particularly suitable for peatland restoration, *Sphagnum* farming, and water remediation. These relationships, although correlative, provide a mechanistic framework linking surface chemistry, biosorption potential, and photosynthetic performance, and identify specific functional groups as key regulators of boundary‐layer chemistry. Future studies should combine high‐resolution measurements of pH, CO_2_ concentration, and photosynthetic activity at the moss–water interface to test causality and resolve spatial heterogeneity at relevant scales.

#### Morphological and physiological traits support CO_2_ uptake under waterlogging

Despite methodological differences, assimilation rates observed here align with published values (Haraguchi *et al*., 2003; Bengtsson *et al*., 2016; Jassey & Signarbieux, 2019), indicating that waterlogging did not impose significant limitations under the hydration conditions applied in this study. The occurrence of bryophytes in habitats with pH > 7 indicates CO_2_‐acquisition under alkaline conditions (Glime & Vitt, 1984). Beyond buffering capacity and chemical reactivity of moss surfaces, additional mechanisms likely contribute to sustaining photosynthesis under aquatic conditions. Morphological traits further modulate CO_2_ uptake. Aquatic taxa with thinner leaves, loosely arranged foliage, and exposed photosynthetic cells reduce water‐layer resistance to CO_2_ uptake, supporting photosynthesis under saturated conditions (Silvola, 1991; Rice & Schuepp, 1995). Here, aquatic/hollow species *S. cuspidatum*, *S. balticum*, and *S. tenellum*, despite surface chemistry allowing acidification, perform less efficiently under neutral‐alkaline conditions but can equal or exceed nonaquatic species in acidic waters. Additional mechanisms, including air retention in hyalocysts, CO_2_‐concentrating strategies, and features poorly characterized in *Sphagnum*, such as cuticular‐like components, further enhance CO_2_ availability (Proctor, 1984; Peñuelas, 1985; Raven *et al*., 1998; Salminen *et al*., 2018; Li & Chang, 2021).

## Competing interests

None declared.

## Author contributions

ADP lead the conceptualization and design of the work, experimental work, formal analysis, and data curation, and wrote, review, and edited the original draft. EP set up the experimental system, supported the implementation photosynthesis experiment, and curated and discussed the gas exchange. AGG and OSP supported the chemical data analysis and interpretation. RR cultivated the mosses in photobioreactors and reviewed the drafts. JMG reviewed the drafts and provided the language editing. FC implemented the data analysis and interpretation, and reviewed the draft. NK supported the interpretation of chemical data. CC provided the supervision and funding acquisition. All authors contributed to review the original draft and have approved the final manuscript.

## Disclaimer

The New Phytologist Foundation remains neutral with regard to jurisdictional claims in maps and in any institutional affiliations.

## Supporting information


**Fig. S1** Amount of binding sites of the field‐collected 20 *Sphagnum* species.
**Fig. S2** Curves of NPQ and *F*_v_′/*F*_m_′ as function of PAR, for 5 *Sphagnum* species collected in the field and four pH values.
**Methods S1** Calculation of binding sites by LPM.
**Table S1** *Sphagnum* species used for the experiments, with the collection areas, habitats, and growth form.
**Table S2** Pearson correlations between amount of binding sites and photosynthesis parameters for the five selected *Sphagnum* spp.
**Table S3** Generalized linear models testing the effects of species‐specific binding sites, pH, and photosynthetic parameters.
Please note: Wiley is not responsible for the content or functionality of any Supporting Information supplied by the authors. Any queries (other than missing material) should be directed to the *New Phytologist* Central Office.

## Data Availability

The data that support the findings of this study are available in the supplementary material of this article: Figs S1, S2, Methods S1 and Tables S1–S3.
