# Supplementary material for: Linking biosurface reactivity and photosynthesis to investigate Sphagnum mosses as ecosystem engineers
Source: New Phytol. 2026 Aug 7;252(1):69–86. doi: 10.1111/nph.71447 (PMC13539958; doi:10.1111/nph.71447)
Supplement: Supplementary file 1 [file NPH-252-69-s001.docx]

## *New Phytologist* Supporting Information

Article title: Linking biosurface reactivity and photosynthesis to investigate *Sphagnum* mosses as ecosystem engineers.

Authors: Anna Di Palma, Emanuele Pallozzi, Aridane G. González, Oleg S. Pokrovsky, Ralf Reski, Janice M. Glime, Fiore Capozzi, Norbert Kavasi, Carlo Calfapietra

Article acceptance date: 28 June 2026

The following Supporting Information is available for this article:

**Fig. S1** Amount of binding sites of the field-collected 20 *Sphagnum* species.

**Fig. S2** Curves of NPQ and F_v_'/F_m_' as function of PAR, for 5 *Sphagnum* species collected in the field and four pH values*.*

**Table S1** *Sphagnum* species used for the experiments, with the collection areas, habitats, and growth form.

**Table S2** Pearson correlations between amounts of binding sites and photosynthesis parameters for the 5 selected *Sphagnum* spp.

**Table S3** Generalized Linear Models testing the effects of species-specific binding sites, pH, and photosynthetic parameters.

**Methods S1** Calculation of binding sites by LPM.

**Fig. S1** Amount of binding sites (mmol g^-1^; *n* = 3) of the 20 *Sphagnum* species collected from nature, computed from acid-base titration curves by applying LPM model. Data are shown as total values and for each type of binding site (C = carboxyl; Pd =phosphodiester; P = phosphoryl; A = amine), and distinguished for moss species (a), subgenus (b) and section (c). SA = *S. angustifolium*; SAO = *S.* *aongstroemii*; SAU = *S. auriculatum*; SB = *S. balticum*; SC = *S. centrale*; SCO = *S. compactum*; SD = *S. divinum*; SF = *S. fimbriatum*; SG = *S.* *girgensohnii*; SM = *S. medium*; SP = *S. palustre*; SPA = *S.* *papillosum*; SQ = *S.* *squarrosum*; SS = *S.* *strictum*; SSN = *S. subnitens*; SSS = *S. subsecundum*; SCU = *S.* *cuspidatum*; ST = *S. tenellum*; STE = *S. teres*; SW = *S. wulfianum*.

**Fig. S2** Curves of NPQ (Non Photochemical Quenching) and F_v_'/F_m_' (maximum efficiency of PSII photochemistry in the light-adapted state) as function of PAR (Photosynthetically Active Radiation; µmol m^-2^ s^-1^), for 5 *Sphagnum* species collected in the field and four pH values (*n* = 5)*.* Values are given with standard errors. Different letters indicate significant differences among pH treatments within each species according to ANCOVA + Tukey’s *post-hoc* test (*p* < 0.05)*.* For NPQ: SA (pH 9 *vs* all, *p* < 0.0001), SB (pH 9 *vs* all, *p* < 0.0001; pH 5 *vs* pH 7, *p* = 0.0022), SG (pH 9 *vs* all, *p* < 0.0001; pH 5 *vs* pH 9, *p* = 0.0398), SS (pH 3 *vs* pH 7, *p* < 0.0001; pH 9 *vs* pH 7, *p* < 0.0001). For F_v_´/F_m_´: SA (pH 9 *vs* all, *p* < 0.0001), SG (pH 3 *vs* pH 9, *p* = 0.0005), SS (pH 3 *vs* pH 5, *p* = 0.0132; pH 5 *vs* pH 7, *p* = 0.0017; pH 5 *vs* pH 9, *p* = 0.0202).

**Table S1** *Sphagnum* species used for the experiments, along with the collection areas, habitats, and growth form (Laine *et al.*, 2018). *Other descriptions from Daniels & Eddy (1985). C. = central; S. = southern; N. = northern; W. =western.

| **Species** | **Area** | **Habitat, Ecology and growth form in collection area** |
| --- | --- | --- |
| ***Acutifolia* subg.** | | |
| ***Squarrosa* sect.** | | |
| *S. squarrosum* | C. Finland | Minerotrophic habitats. Shade-tolerant mats and small cushions. Most common in coniferous fens and drier surfaces than for *S. teres*. *Slightly drier, raised ground / hummocks. |
| *S. teres* | C. Finland | Minerotrophic habitats. Sparse to dense mats in wet open and wooded fens. *Tolerance of a wide range of variation in trophic status, water level and shade. |
| ***Polyclada* sect.** | | |
| *S. wulfianum* | C. Finland | Minerotrophic habitats. Shade tolerant, loose mats and small cushions in drier spruce-dominated mires. *Typically, in moist boreal forest margins, small hummocks, not in deep water. |
| ***Insulosa* sect.** | | |
| *S. aongstroemii* | N. Finland | Minerotrophic habitats. Alluvial or wooded fens, along brooks and rivers. Dense cushions and mats. *Often in wetter lawn or hollow-like microtopography. |
| ***Acutifolia* sect.** | | |
| *S. fimbriatum* | C. Finland | Minerotrophic habitats. Soft hummocks and carpets in flooded open or wooded mires and forests. *Typically, on slightly raised ground (hummocks) or in damp woodland; mesotrophic fens. |
| *S. girgensohnii* | S. Finland | Minerotrophic habitats. Shade-tolerant, loose mats and small cushions in moist spruce-forests or medium-rich fens. *Often in shaded, moist woodland or edges of mires, above water table. |
| *S. subnitens* | C. Finland | Strongly minerotrophic habitats. Individual shoots or small cushions in lawns and carpets. *In wetter lawns and pools; often in transitional microhabitats. |
| ***Cuspidata* subg.** | | |
| *S. angustifolium* | S. Finland | Wide tolerance from ombrotrophic to intermediate minerotrophic conditions. From hummocks to lawns, with tolerance to moderate shading. *Often associated with hummocks; tolerates flushes and mineral-enriched bogs. |
| *S. balticum* | S.W. Finland | Ombrotrophic to minerotrophic habitats. Carpets and lawns of open mires. Typical in the margin of raised bog hollows and pools. *In wet, oligotrophic to slightly mesotrophic areas. |
| *S. cuspidatum* | S. Finland | Ombrotrophic to oligotrophic habitats. Commonly in hollows and pools. *Aquatic / floating‑mat species in pool or hollow communities. |
| *S. tenellum* | S. Finland | Ombrotrophic to weakly minerotrophic habitats. Single shoots or loose cushions typically in small patches of mire surfaces. *Often in relatively wet, fen‑type microhabitats (raised but water‐influenced). |

**Table S1** Continued.

| **Species** | **Area** | **Habitat, Ecology and growth form in collection area** |
| --- | --- | --- |
| ***Rigida* subg.** | | |
| *S. compactum* | S. Finland | Low, compact cushions in shaded, periodically dry, oligotrophic habitats. In water flushes of rocky habitats as well. *Wetter lawns / microtopographic depressions. |
| *S. strictum* | C. Sweden | Lose mats or low compact tussocks in ombrotrophic bogs. Margin of treed fens, blanket mires, wet moorlands and moist heaths. *Strongly hummock-forming, on drier raised hummocks. |
| ***Sphagnum* subg.** | | |
| *S. centrale* | C. Finland | Hummocks and dense carpets in mesotrophic habitat, often in shaded condition of wooded or herb rich fens. *Found in mid- to low-hummock microtopography. |
| *S. divinum* | C. Finland | Minerotrophic treed fens. Scarcely wet substrate. *Hollow‑lawn species (wet carpets). |
| *S. medium* | C. Finland | Low hummocks, lawns and carpets of ombrotrophic habitats. |
| *S. palustre* | S.W. Finland | Loose hummocks and dense carpets in mesotrophic habitat, often in shaded condition of wooded fens. *Wide ecological amplitude; common in lawns and hummocks. |
| *S. papillosum* | C. Finland | Low hummocks and dense carpets in weakly minerotrophic habitat. Common from oligotrophic to mesotrophic sites. *Hummock-forming species, often on raised bog mounds. |
| ***Subsecunda* subg.** | | |
| *S. auriculatum* | S.W. Finland | Oligotrophic habitats. Can grows in carpets, floating or submerged. *In more mesic lawns / wetter carpets. |
| *S. subsecundum* | S. Finland | Carpets as loose mats in mesotrophic habitat. *Common in hollows / aquatic mats. |

**Table S2** Pearson correlations between amounts of binding sites (BS; total and by type) and photosynthesis parameters. C-Pd = carboxyls-phosphodiesters; C= carboxyls; P = phosphoryls; A = amines; *r* = correlation coefficient; *r^2^*= coefficient of determination; *A_max_* = maximum photosynthetic rate; *Φ* = Apparent quantum yield; LCP = Light compensation point; R_d_ = dark respiration.

|  | **pH** | **Total BS** | | **C-Pd** | | **C** | | **P** | | **A** | |
| --- | --- | --- | --- | --- | --- | --- | --- | --- | --- | --- | --- |
|  |  | ***r*** | ***r^2^*** | ***r*** | ***r^2^*** | ***r*** | ***r^2^*** | ***r*** | ***r^2^*** | ***r*** | ***r^2^*** |
| ***A_max_*** | 3 | 0.14 | *0.02* | 0.74 | *0.54* | 0.01 | *0.00* | -0.21 | *0.04* | 0.76 | *0.57* |
|  | 5 | 0.69 | *0.47* | 0.78 | *0.61* | -0.02 | *0.00* | 0.57 | *0.33* | 0.46 | *0.21* |
|  | 7 | 0.72 | *0.52* | 0.78 | *0.60* | -0.17 | *0.03* | 0.67 | *0.44* | 0.11 | *0.01* |
|  | 9 | -0.49 | *0.24* | -0.42 | *0.18* | -0.73 | *0.53* | -0.16 | *0.02* | 0.31 | *0.10* |
| ***Φ*** | 3 | -0.13 | *0.02* | 0.14 | *0.02* | 0.13 | *0.02* | -0.55 | *0.30* | 0.74 | *0.54* |
|  | 5 | -0.02 | *0.00* | 0.73 | *0.53* | 0.04 | *0.00* | -0.37 | *0.14* | 0.94 | *0.89* |
|  | 7 | -0.07 | *0.01* | 0.07 | *0.00* | 0.13 | *0.02* | -0.45 | *0.21* | 0.41 | *0.17* |
|  | 9 | -0.19 | *0.04* | 0.07 | *0.00* | 0.04 | *0.00* | -0.50 | *0.25* | 0.94 | *0.89* |
| **LCP** | 3 | -0.01 | *0.00* | -0.06 | *0.00* | 0.83 | *0.68* | -0.78 | *0.61* | 0.15 | *0.02* |
|  | 5 | -0.46 | *0.21* | -0.48 | *0.23* | 0.46 | *0.22* | -0.73 | *0.53* | -0.01 | *0.00* |
|  | 7 | -0.65 | *0.42* | -0.66 | *0.44* | 0.14 | *0.02* | -0.88 | *0.78* | 0.05 | *0.00* |
|  | 9 | -0.42 | *0.17* | -0.62 | *0.39* | -0.10 | *0.01* | -0.71 | *0.50* | 0.66 | *0.44* |
| **R_d_** | 3 | 0.42 | *0.18* | 0.52 | *0.27* | -0.31 | *0.10* | 0.85 | *0.72* | -0.71 | *0.50* |
|  | 5 | 0.55 | *0.31* | 0.77 | *0.60* | -0.11 | *0.01* | 0.89 | *0.80* | -0.63 | *0.39* |
|  | 7 | 0.49 | *0.24* | 0.52 | *0.27* | -0.15 | *0.02* | 0.87 | *0.76* | -0.36 | *0.13* |
|  | 9 | 0.29 | *0.09* | 0.60 | *0.36* | 0.07 | *0.00* | 0.57 | *0.33* | -0.83 | *0.69* |

| **Table S3** Generalized Linear Models (GLMs) and Tukey’s Honestly Significant Difference (HSD) *post-hoc* test (*p* < 0.05) applied to test the overall effects of moss species identity (proxy for binding sites), pH, and their interaction on *Sphagnum* photosynthetic parameters. *R²* indicates the proportion of explained variance. *A_max_* = maximum photosynthetic rate; *Φ* = apparent quantum yield; LCP = Light compensation point; R_d_ = dark respiration. | | | |
| --- | --- | --- | --- |
| **Parameter** | ***F**** | ***p**** | ***R^2^*** |
| *A_max_* | 3.2 | < 0.001 | 0.43 |
| *Φ* | 4.5 | < 0.001 | 0.52 |
| LCP | 4.3 | < 0.001 | 0.5 |
| R_d_ | 14.6 | < 0.001 | 0.77 |
| **F- and p-values referred to the overall significance. df = 19.* | | | |
| **Species** | **Parameter** | **Tukey’s HSD Contrasts** | ***p*** |
| *S. balticum* | *A_max_* | pH 3 *vs* pH 9 | 0.030 |
|  |  | pH 5 *vs* pH 9 | 0.010 |
|  |  | pH 7 *vs* pH 9 | 0.020 |
|  | LCP | pH 5 *vs* pH 9 | 0.019 |
|  |  | pH 7 *vs* pH 9 | 0.022 |
|  | R_d_ | pH 5 *vs* pH 9 | 0.041 |
|  |  | pH 7 *vs* pH 9 | 0.004 |
| *S. girgensohnii* | *Φ* | pH 3 *vs* pH 9 | 0.040 |
|  |  | pH 5 *vs* pH 7 | 0.017 |
|  |  | pH 7 *vs* pH 9 | 0.008 |
|  | LCP | pH 3 *vs* pH 9 | 0.040 |

**Methods S1**

LPM uses a grid of fixed p*K* values and optimizes the binding site concentrations. In each case, zero is considered as a possible solution then, a p*K* spectrum is produced for the discrete binding sites computed by the number of p*K* values. The acid-base titration experiments can be modeled according with the proton dissociation reaction for a single protonated site:

where *HL* are the protonated binding sites on the moss surface and *H^+^* is the hydrogen concentration measured directly with a pH electrode. *L^-^* is the deprotonated surface reactive site with a net negative charge. Accordingly, the apparent proton dissociation constant is:

$$K_{a}=\frac{\left[L^{-}\right]\left[H^{+}\right]}{\left[HL\right]}$$

where p*K_a_*=-log_10_*K_a_*. Therefore, the experimental net surface charge excess for the *i*th addition of titrant (*b_meas,i_*) is:

$$b_{meas,i}=C_{bi}-C_{ai}+\left[H^{+}\right]_{i}-\left[OH^{-}\right]_{i}$$

where *C_ai_* and *C_bi_* are the acid base concentrations at the *i*th addition of titrant. The calculated net surface charge excess can be written as:

Here, *m* is the number of binding sites and [*L_T_*] is the site density. *K_a,j_* is the apparent acidity constant for the *j*th site, and *S_0_* is a constant which is necessary in order to account for positive changes on the surface. In the case of pH-edge adsorption, within the formalism of *K_s,j_*, the reaction between a metal in solution and available binding sites on the moss cell surface is defined as:

where *Me^2+^* represents a generic metal divalent cation, *B_j_* is a surface reactive site and *K_s,j_* is the apparent concentration equilibrium constant conditional on ionic strength. For a *j-*th deprotonated binding sites at the *i-*th pH values, *K_s,j_* is:

$$K_{s,j}=\frac{\left[MeB_{j}^{+}\right]_{i}\left[H^{+}\right]_{meas,i}}{\left[Me^{2+}\right]_{meas,i}\left[B_{j}H^{0}\right]_{i}}$$

where *K_s,j_* is a function of experimentally measured proton and metal concentrations ([H^+^]*_meas,i_* and [*Me^2^*^+^]*_meas,i_*) and the amount of Me^2+^ bound to the *j-*th site at the *i-*th pH value ([*MeB_j_*^+^]*_i_*). In the case of Langmuirian isotherm adsorption (fixed pH), the LPM considers the reaction between metal and binding sites expressed as:

where *B_j_* is the specific surface functional group and *K_m,j_* is the apparent metal-ligand binding constant conditional on ionic strength, for a *j-*th deprotonated functional group at fixed pH value is written as:

$$K_{m,j}=\frac{\left[MeB_{j}^{+}\right]_{i}}{\left[Me^{2+}\right]_{meas,i}\left[B_{j}\right]_{i}}$$

*K_m,j_* is a function of the experimental metal concentration ([*Me^2+^*]*_meas,i_*) and of the amount of Me^2+^ bound to the *j-*th site as a function of increasing biomass and at fixed pH value ([*MeB_j_^+^*]*_i_*). As described for pH-edge data, the available binding sites on moss surfaces are computed and assigned to a fixed p*K_m,j_* grid. In addition, the values of *K_s,j_* and *K_m,j_* computed cannot be directly comparable because K_s_ is a function of *K_m_* (*K_s_*= *K_m_*·*K_a_*), where *K_a_* is the acidity constant for a specific functional group on the moss surface.

**References**

**Daniels RE, Eddy A. 1985**. *Handbook of European Sphagna.* Institute of Terrestrial Ecology, Natural Environment Research Council. ISBN 0904282821.
